# Digital Footprint of Academic Vascular Surgeons in the Southern United States on Physician Rating Websites: Cross-sectional Evaluation Study

**DOI:** 10.2196/22975

**Published:** 2021-02-24

**Authors:** Qi Yan, Katherine J Jensen, Rose Thomas, Alyssa R Field, Zheng Jiang, Christian Goei, Mark G Davies

**Affiliations:** 1 Division of Vascular Surgery Department of Surgery UT Health San Antonio San Antonio, TX United States; 2 Department of Surgery UT Health San Antonio San Antonio, TX United States; 3 Department of Surgery Shanghai Medical College Fudan University China; 4 South Texas Center for Vascular Care San Antonio, TX United States

**Keywords:** internet, patient satisfaction, quality of care, physician rating sites, patient experience, professional reviews, social media

## Abstract

**Background:**

The internet has become a popular platform for patients to obtain information and to review the health care providers they interact with. However, little is known about the digital footprint of vascular surgeons and their interactions with patients on social media.

**Objective:**

This study aims to understand the activity of academic vascular surgeons on physician rating websites.

**Methods:**

Information on attending vascular surgeons affiliated with vascular residency or with fellowships in the Southern Association for Vascular Surgery (SAVS) was collected from public sources. A listing of websites containing physician ratings was obtained via literature reviews and Google search. Open access websites with either qualitative or quantitative evaluations of vascular surgeons were included. Closed access websites were excluded. Ranking scores from each website were converted to a standard 5-point scale for comparison.

**Results:**

A total of 6238 quantitative and 967 qualitative reviews were written for 287 physicians (236 males, 82.2%) across 16 websites that met the inclusion criteria out of the 62 websites screened. The surgeons affiliated with the integrated vascular residency and vascular fellowship programs in SAVS had a median of 8 (IQR 7-10) profiles across 16 websites, with only 1 surgeon having no web presence in any of the websites. The median number of quantitative ratings for each physician was 17 (IQR 6-34, range 1-137) and the median number of narrative reviews was 3 (IQR 2-6, range 1-28). Vitals, WebMD, and Healthgrades were the only 3 websites where over a quarter of the physicians were rated, and those rated had more than 5 ratings on average. The median score for the quantitative reviews was 4.4 (IQR 4.0-4.9). Most narrative reviews (758/967, 78.4%) were positive, but 20.2% (195/967) were considered negative; only 1.4% (14/967) were considered equivocal. No statistical difference was found in the number of quantitative reviews or in the overall average score in the physician ratings between physicians with social media profiles and those without social media profiles (departmental social media profile: median 23 vs 15, respectively, *P*=.22; personal social media profile: median 19 vs 14, respectively, *P*=.08).

**Conclusions:**

The representation of vascular surgeons on physician rating websites is varied, with the majority of the vascular surgeons represented only in half of the physician rating websites The number of quantitative and qualitative reviews for academic vascular surgeons is low. No vascular surgeon responded to any of the reviews. The activity of vascular surgeons in this area of social media is low and reflects only a small digital footprint that patients can reach and review.

## Introduction

Social media resources continue to be used by commercial and nonprofit organizations for social marketing because of the advantages of access to a large number of consumers, presence of transparency, the wide global reach, the ability to boost web page traffic, and the ability to promote the brand name [1]. With reference to health care, social media tools play an essential role in reputation management, public outreach, health promotion, and patient education [2]. An additional tool is the provision of quantitative and qualitative ratings of physician performance on physician rating websites that can be shared publicly. Seventy percent of the top 10 Google search results on specific physicians are third-party websites such as physician rating websites [3].

Physician rating websites display valuable information for a consumer regarding a physician’s practice, including the physician’s area of expertise, office location, office hours, insurance accepted, in addition to quantitative and qualitative reviews from past consumers. Although surveys have indicated that the insurance accepted, referrals from primary care physicians, and reputation are important in the selection of physicians, 65% of the physician rating website users have been shown to choose a physician after viewing positive reviews on websites, and conversely, 52% of the physician rating website users have avoided providers owing to the negative reviews shown on the websites [4,5].

Although physician rating websites contain a broad range of physician-specific data, the physicians rated on these websites are not evenly distributed among medical specialties [6-16]. While orthopedic surgeons are well represented on physician rating websites with over 90% of the surgeons having ratings on the most popular physician rating websites, radiologists, who have limited direct face-to-face contact with patients, have a very small digital physician rating website footprint with only 20% rated on any of the 5 physician rating websites studied [7-9,11,13]. Multiple studies have demonstrated differences in the ratings across multiple specialties. Physicians in the fields of cardiac surgery, nephrology, genetics, and radiology receive higher ratings, whereas those in addiction medicine, dermatology, neurology, and psychiatry receive lower ratings [16]. Vascular surgery is emerging as a distinct subspecialty and is currently undergoing a branding and identity campaign. In light of this change in the perception of this specialty, there exists a knowledge gap on the digital footprint and performance of vascular surgeons across the spectrum of physician rating websites. Understanding this gap can offer this specialty a roadmap to improve public perception and will prompt further research on the effect of branding and marketing on the ratings within the physician rating websites for vascular surgery. The aim of this study was to examine the accuracy of professional demographics, the presence and responsiveness of academic vascular surgeons across open access physician rating websites, and the quantity and quality of patient reviews within a defined geographic region. The aim was also to define the digital physician rating website footprint of vascular surgery to ascertain whether academic vascular surgeons have evolved to embrace and participate in these reviews.

## Methods

This is a cross-sectional study of publicly held data in search engines and websites accessed from a US internet service provider from September 2019 to November 2019. Websites containing physician ratings were obtained via literature reviews and Google search. This study examines data in the public domain and does not contain Health Insurance Portability and Accountability Act (HIPAA) information or interactions with individuals; thus, it is exempt from institutional review board approval or consent.

The current integrated vascular residency and vascular fellowship program lists were obtained from the Accreditation Council for Graduate Medical Education website. Attending vascular surgeons affiliated with each of these programs in the Southern Association for Vascular Surgery (SAVS) were collected [17]. Websites reporting physician ratings were obtained via literature reviews and Google search by using the terms “rate doctors,” “MD review,” “physician ranking,” “doctor rating,” “find doctors,” and “best doctors.” The inclusion criteria were as follows: (1) open access websites and (2) websites that allowed qualitative or quantitative reviews by patients. The exclusion criteria were as follows: (1) physician rating websites outside of the United States; (2) websites limited to a certain geographic area in the United States; (3) websites that excluded vascular surgeons; (4) websites linked to a health care system, and (5) websites that were inaccessible.

Health care review websites obtain the physician list through public records from the National Provider Identifier Registry, medical boards, etc. General review websites allow users or business owners to add physicians or edit information, whereas health care review websites require changes to be made through the management team. Most websites allow physicians to claim their profile for free after they create an account and go through the prompted steps. These websites then allow physicians to manage their profiles, audit for accuracy, respond to reviews, and dispute reviews depending on the website. Some websites offer sponsored profiles to physicians for a fee to promote their practice. Multimedia Appendix 1 provides the definitions of the different profiles of the physicians.

Data collection was performed between August 2019 and September 2019. Physician search was performed on individual websites with physician first name/first initial, middle name/middle initial, last name, and location in various combinations and orders. Supplementary Google search was performed with “physician name, website domain name” to enhance the discovery of the physician’s profile. A similar strategy was used in finding physician or department profiles on social media websites such as Facebook, Twitter, and LinkedIn. Physician-specific information such as gender, age, educational background, training, and professional association was collected through their professional websites affiliated with their institutions and physician rating websites.

Most physician rating websites used a ranking score of 1-5 while Dr.Score and Healthcare reviews used a ranking score of 1-10; the scores of these 2 sites were converted to the standard 5-point scale (1-5) for comparison. Physician rating websites with more than 1000 quantitative reviews were used to examine correlation. The Kendall rank correlation coefficient was used to adjust for ties. USNews&World Report was excluded from this analysis because the ratings in this website were derived from over 100 unspecified web-based sources gathered by a private company and does not collect patient reviews or ratings directly. Descriptive data were presented as median (IQR). Kendall correlation coefficient was used to evaluate the correlation between continuous variables. Mann-Whitney *U* or Kruskal-Wallis test with post hoc Wilcoxon test was used for continuous data. Chi-square test was used to compare categorical data with a Bonferroni adjustment used for all post hoc tests with adjusted *P* values reported. Data analysis was performed in RStudio version 1.2.5001 (RStudio Inc).

## Results

### Review of the Commercial Websites

**Figure 1:**
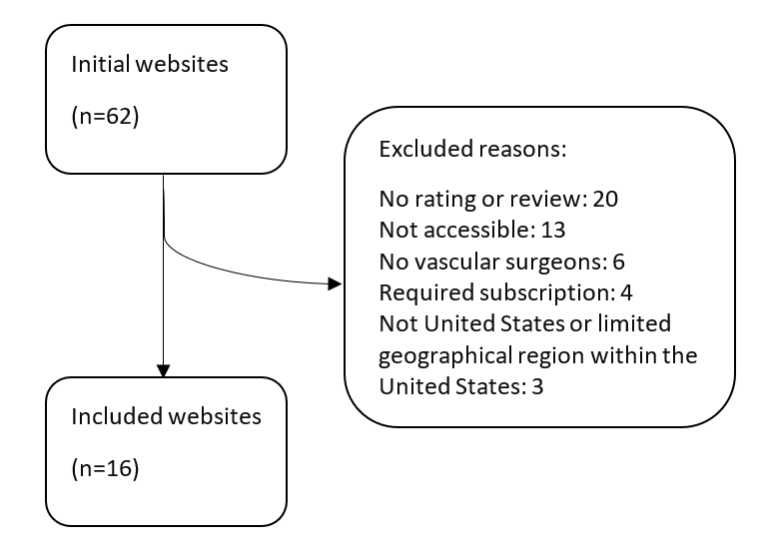
Flow diagram for website inclusion.

Sixteen out of 62 websites met the inclusion criteria (Multimedia Appendix 2). Figure 1 shows the flow diagram of the websites included and excluded in this study. WebMD and USNews&World Report were the 2 sites that listed the most number of physicians (271/287, 94.4%) while Yellowbot (48/287, 16.7%) had the least number of physicians (Table 1). Websites had specific search strategies that provided higher yield; however, none of the websites provided instructions. Table 2 shows the search strategy on the 4 most common physician rating websites. Sixteen physician rating websites were included after screening 62 initial websites.

**Table 1:** Physician profiles across 16 physician rating websites (N=287).

Websites	Physicians with profile, n (%)^a^	Incomplete profiles, n (%)^b^	Inaccurate profiles, n (%)^c^
WebMD	271 (94.4)	25 (9.6)	27 (10.0)
USNews&World Report	271 (94.4)	1 (0.4)	11 (4.1)
Caredash	266 (92.7)	7 (2.6)	21 (7.9)
Vitals	264 (92.0)	3 (1.1)	19 (7.2)
Healthgrades	264 (92.0)	24 (9.1)	44 (16.7)
YP.com	209 (72.8)	86 (41.1)	23 (11.0)
RateMD	199 (69.3)	156 (78.4)	50 (25.1)
Dr.Score	147 (51.2)	46 (31.3)	28 (19.0)
Insiderpages	101 (35.2)	101 (100.0)	0 (0.0)
Local	90 (31.4)	87 (96.7)	0 (0.0)
Zocdoc	79 (27.5)	11 (13.9)	4 (5.1)
Yellowbook	74 (25.8)	72 (97.3)	0 (0.0)
Healthcare reviews	70 (24.4)	22 (31.4)	0 (0.0)
Wellness	62 (21.6)	26 (41.9)	17 (27.4)
Yelp	53 (18.5)	53 (100.0)	10 (18.9)
Yellowbot	48 (16.7)	46 (95.8)	0 (0.0)

^a^Total number of physicians with profiles on the 16 websites=286.

^b^Profiles (n=766) with less than 50% of the required information (eg, training, expertise) or profiles that lack any physician-specific information besides practice location and office contact information. Percentage was calculated as the number of physicians with incomplete profiles by the total number of physicians with profiles for each website.

^c^Profiles with any inaccuracy in physician practice and demographic information (n=252). Percentage was calculated as the number of physicians with inaccurate profiles by the total number of physicians with profiles for each website.

**Table 2:** Search strategy on the 4 most common physician rating websites (N=287).

Website name	Name search	Location search	Required search on Google, n (%)
Vital	Must use middle initial, does not have full middle name in the system. If first name in the system is also initial only, entering full name will not find the physician.	Has a certain degree of matching by name during the search regardless of the correct location.	0 (0.0)
Healthgrades	Name must have the exact match being “first name, last name” in order to find through search. However, will provide matching in the search box without such restriction.	Has a certain degree of matching by name in the search box regardless of the correct location.	7 (2.4)
WebMD	Good name match. No requirement for the order of the name or differentiation between initial versus expanded name.	Location needs to be correct. Very low degree of matching by name only.	18 (6.3)
USNews&World Report	Must have correct expanded first name and last name. Order of the name and the middle name does not affect search.	Match of name is sufficient, correct location not required.	1 (0.3)

### Review of the Academic Divisional Participation

One potential confounding factor in individual physician profiles is the corresponding activity of the divisional profile digital footprint. Nine (9/37, 24%) vascular surgery divisions had social media profiles: 7 had only Twitter accounts, 1 had only Facebook profiles, and 1 had both Twitter and Facebook profiles. Physicians in the institutions with established departmental social media websites had a higher number of ratings compared to those without established departmental social media websites (median 23 [IQR 5-38] vs 15 [IQR 7-33], respectively, *P*=.22), although this was not statistically significant.

### Review of Individual Physician Participation

Surgeons affiliated with the integrated vascular residency and vascular fellowship program of SAVS had a median (IQR) of 8 (7-10) profiles across 16 websites with only 1 surgeon having no web presence on any of the sites. Most physician profiles (2214/2466, 89.8%) were accurate, reflecting correct demographic affiliations and practice information (Table 1). Of the 8 websites wherein claimed profiles were clearly distinguished, only 14.9% (43/287) of the physicians considered in this study had at least one claimed profile. Of the single website that had clear notations of the sponsored profiles on the profile page itself, no vascular surgeon had sponsored profiles. Of the 287 physicians, 115 (40.1%) were members of the Society of Vascular Surgery and 82 (28.6%) were members of other vascular societies, while the remainder did not disclose their affiliations; 195 (67.9%) physicians had profiles on at least one social media platform: 57.5% (165/287) on LinkedIn, 19.9% (57/287) on Twitter, and 18.5% (53/287) on Facebook.

A total of 6238 ratings and 967 narrative reviews were written for 287 physicians (236 males, 82.2%) affiliated with the integrated vascular residency and vascular fellowship program within the SAVS across the 16 websites surveyed (Table 3 and Table 4). The median number of quantitative ratings for each physician among those with at least 1 rating was 17 (IQR 6-34, range 1-137) and the median number of narrative reviews among those with at least 1 narrative review was 3 (IQR 2-6, range 1-28); 12.9% (37/287) of the physicians had 0 quantitative reviews and 31.0% (89/287) had 0 qualitative reviews. Ratings were overwhelmingly positive, with a median weighted average score of 4.4 (IQR 4.0-4.9) out of a total score of 5. Most narrative reviews (758/967, 78.4%) were also positive, but 20.2% (195/967) of them were considered negative; only 1.4% (14/967) were considered equivocal. Physicians with negative narrative reviews had lower ratings compared to those without (median 4.07 vs 4.7, *P*=.001). There was no physician response to any patient review.

**Table 3:** Physician rating scores across 16 physician rating websites (N=287).

Websites	Physicians with rating, n (%)^a^	Total quantitative ratings (n=6238), n	Median score	IQR
Vitals	217 (75.7)	1731	4.4	4-5
WebMD	200 (69.7)	1737	4.5	4-5
Healthgrades	193 (67.2)	1193	4.6	3.7-5
USNews&World Report	71 (24.7)	1296	5	4-5
RateMD	46 (16.0)	103	4	3-5
Dr.Score	24 (8.3)	34	5	4-5
Caredash	18 (6.3)	22	5	5-5
Wellness	11 (3.8)	20	4.5	3.8-5
Yellowbot	7 (2.4)	17	5	5-5
Zocdoc	7 (2.4)	65	5	4-5
Insiderpages	6 (2.1)	13	5	4.3-5
YP.com	4 (1.4)	4	5	4-5
Yelp	2 (0.7)	2	3	2-4
Healthcare reviews	1 (0.3)	1	5	N/A^b^
Local	0 (0.0)	0	N/A	N/A
Yellowbook	0 (0.0)	0	N/A	N/A

^a^Total number of physicians with at least 1 rating=251.

^b^N/A: not applicable.

**Table 4:** Narrative reviews across 16 physician rating websites.

Websites	Physicians with narrative reviews (n=287), n (%)^a^	Total narrative reviews (n=967), n	Positive narrative reviews, n (%)^b,c^	Neutral narrative reviews, n (%)^c,d^	Negative narrative reviews, n (%)^c,e^	Patient found useful (n=737), n	Patient found not useful (n=150), n
Vitals	151 (52.6)	482	374 (77.6)	5 (1.0)	103 (21.4)	0	0
WebMD	1 (0.3)	1	1 (100.0)	0 (0.0)	0 (0.0)	0	0
Healthgrades	136 (47.4)	308	248 (80.5)	0 (0.0)	60 (19.5)	704	148
USNews&World Report	0 (0.0)	0	0 (0.0)	0 (0.0)	0 (0.0)	0	0
RateMD	43 (15)	88	58 (65.9)	5 (5.7)	25 (28.4)	29	0
Dr.Score	0 (0.0)	0	0 (0.0)	0 (0.0)	0 (0.0)	0	0
Caredash	14 (4.9)	16	15 (93.8)	1 (6.3)	0 (0.0)	4	2
Wellness	8 (2.8)	17	15 (88.2)	0 (0.0)	2 (11.8)	0	0
Yellowbot	7 (2.4)	19	19 (100.0)	0 (0.0)	0 (0.0)	0	0
Zocdoc	6 (2.1)	29	23 (79.3)	3 (10.3)	3 (0.3)	0	0
Insiderpages	1 (0.3)	1	1 (100.0)	0 (0.0)	0 (0.0)	0	0
YP.com	4 (1.4)	4	3 (75.0)	0 (0.0)	1 (25.0)	0	0
Yelp	2 (0.7)	2	1 (50.0)	0 (0.0)	1 (50.0)	0	0
Health care reviews	0 (0.0)	0	0 (0.0)	0 (0.0)	0 (0.0)	0	0
Local	0 (0.0)	0	0 (0.0)	0 (0.0)	0 (0.0)	0	0
Yellowbook	0 (0.0)	0	0 (0.0)	0 (0.0)	0 (0.0)	0	0

^a^197 physicians received narrative reviews.

^b^Total number of positive narrative interviews=758.

^c^The percentages for the positive, neutral, and negative narrative review columns were calculated over the total narrative reviews in the 3rd column.

^d^Total number of neutral narrative interviews=14.

^e^Total number of negative narrative interviews=195.

### Correlation Between the Rating Scores

The physician scores on Vitals and WebMD correlated well (Kendall τ=0.78, *P*<.001) while those on Healthgrades correlated poorly with Vitals (Kendall τ=0.15, *P*=.007) and WebMD (Kendall τ=0.17, *P*=.006). Years of experience (Kendall τ =–0.12, *P*=.007), personal social media profile (Kendall τ=0.03, *P*=.57), departmental social media profile (Kendall τ=–0.05, *P*=.34), and number of ratings (Kendall τ=–0.14, *P*=.001) did not correlate or they only weakly correlated with the weighted average score.

### Physician and Practice Factors Affecting the Size of the Digital Footprint

**Figure 2:**
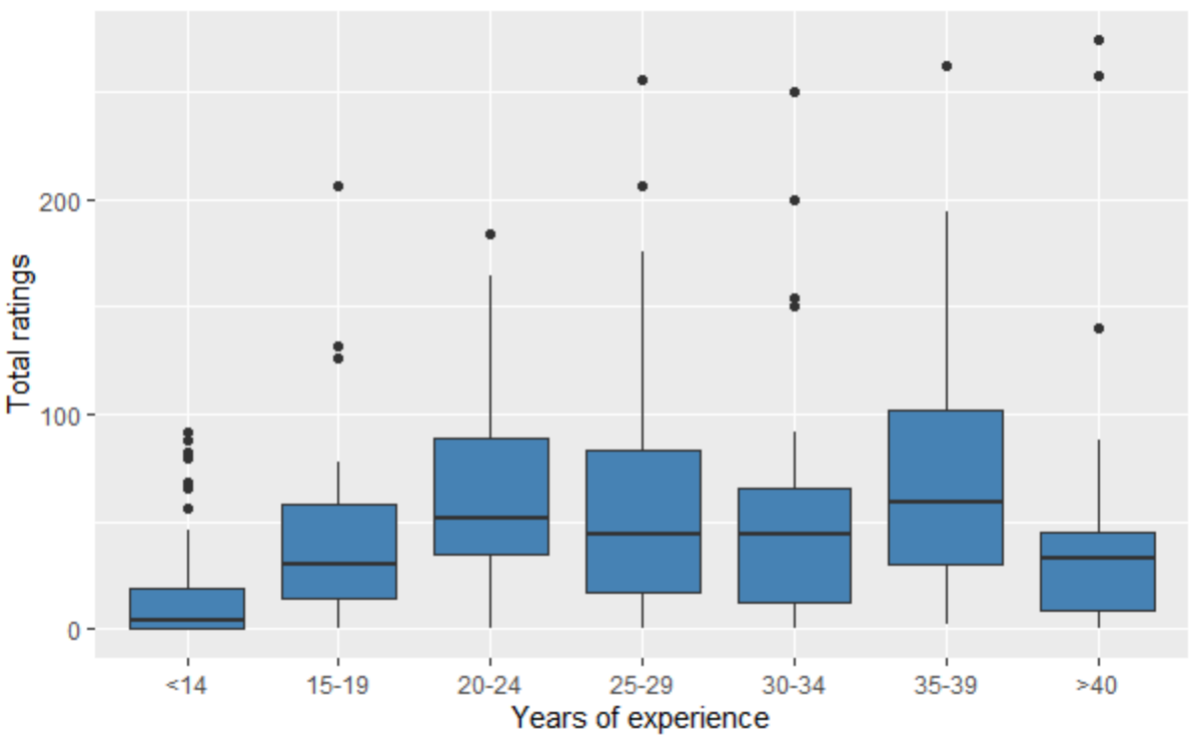
Total number of ratings across 16 physician rating websites by years of experience (number of years after graduation from allopathic or osteopathic school). The lower, middle, and upper hinges of the box represent 25th percentile, 50th percentile or median, and 75th percentile, respectively. Whiskers represent 1.5 interquartile range, and points represent outliers.

**Figure 3:**
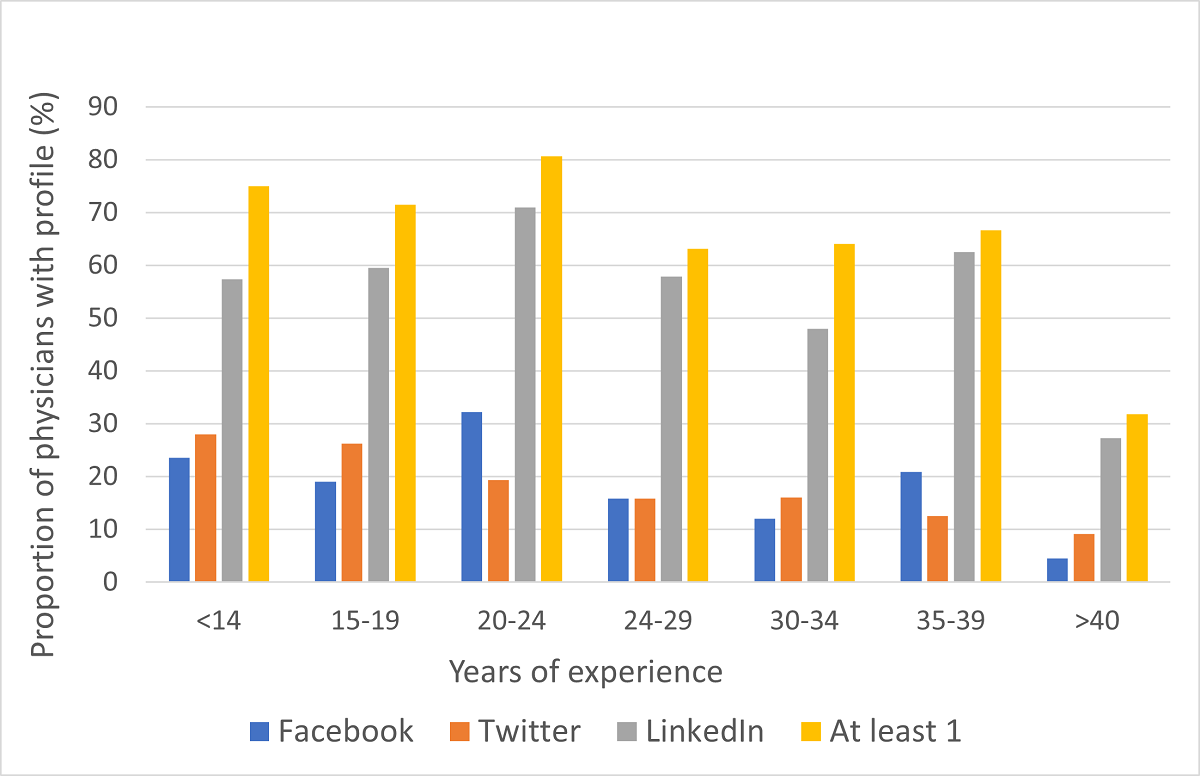
Proportion of physicians with profiles on 3 different social media platforms among physicians with different years of experience by the number of years after graduation from an allopathic or osteopathic school.

The social media profiles of vascular surgeons (especially LinkedIn) and the rating number followed a similar curve with small peaks in the age groups of 20-24 years and 35-39 years (Figure 2 and Figure 3). Physicians with individual social media profiles had a higher median number of ratings compared to those who did not; however, this did not reach statistical significance (19 vs 14, respectively, *P*=.08). Fewer female vascular surgeons had personal social media profiles (*P*=.02). Female surgeons had fewer years of experience (median 14 vs 24 for males, respectively, *P*=.001), fewer profiles (7.5 vs 9, respectively, *P*=.02), fewer number of ratings (median 6 vs 19, respectively, *P*<.001) but a similar number of narrative reviews (3 vs 4, respectively, *P*=.23). Physicians in a practice with 10 vascular surgeons or more had more ratings (21 vs 13, respectively, *P*=.01) but similar number of profiles (median 9 vs 8, respectively, *P*=.09) and narrative reviews (4 vs 3, respectively, *P*=.18).

### Reviewer Information

Of the 2 physician rating websites with a significant number of narrative reviews, Vitals did not provide reviewer names while a third (111/308, 36.0%) of the reviewers on Healthgrades provided their full name; another 29.5% (91/308) provided only the initial or the first name, while a third (106/308, 34.4%) remained completely anonymous.

## Discussion

### Principal Results

Our study found that the presence of academic vascular surgeons affiliated with the integrated vascular residency and vascular fellowship program within the SAVS was low on physician rating websites. Of the 287 physicians, 87.4% (251/287) of the physicians had at least one rating across 16 physician rating websites and 68.6% (197/287) had at least one narrative review. The density of the reviews was low, with a median of 17 for the quantitative ratings and a median of 3 for the narrative reviews for those who had at least 1 rating or narrative review across 16 physician rating websites. No physician responded to any of the reviews.

### Limitations of This Study

The study has a limited sample and may not be extrapolated to all US academic vascular physicians. We were unable to correlate physician ratings with USNews&World Report hospital rankings, urban or rural locations, and whether the academic center had a social media department because minimal variation existed in these variables. It was not possible to identify the type of procedure offered by the physicians to assess its effect on the patient reviews. We did not correlate hospitals’ own review systems with physician rating websites or Press Ganey scores. Additionally, information on the internet is dynamic; physicians could have accumulated additional reviews since the time of our data collection. Lastly, we did an extensive search to investigate the physician rating websites, and multiple searches were performed on individual providers to ensure that we captured maximum information. However, it is possible that there were websites or profiles that we may have missed or we may not have investigated. This likely does not significantly affect our study, as it would be equally difficult for patients to locate such websites or profiles and likely contain minimal information.

### Comparison With Prior Work

Direct comparisons between previous studies of physician rating websites is difficult due to differences in physician selection. There is a general trend of an increasing percentage of physicians rated over time. Vascular surgeons were significantly underrepresented compared to orthopedic surgeons; 94.3%-99.5% of the orthopedic surgeons were rated on physician rating websites with a much higher number of ratings—triple that of vascular surgeons in some instances, and we speculate that this difference may be attributed to differences in the specialties rather than differences in physician demographics [7-9,11,12,18]. The cluster of studies on the digital footprint of orthopedic surgeons reflects better awareness of social media, more effort in the web-based promotion of their practice, and thus, a larger digital footprint [7-9,11,12,18,19].

In general, patients rated vascular surgeons very positively, with the median score for almost all the websites being 4 or higher on a 5-point scale, and 78.4% (758/967) of the comments were positive overall. This is consistent with prior findings regardless of specialty [6,11,20-22]. Despite the overall positivity of the reviews, the consistency of the ratings at the physician level across physician rating websites was variable. Similar to that reported in previous studies, Vitals and WebMD had excellent correlation, but both correlated poorly with Healthgrades [8,11].

In our study, we did not find any physician demographic characteristics, level of social media presence, or total rating frequency that contributed to the overall rating scores. Some studies found that younger physicians received higher scores, which could be attributed to the better relationships between younger patients and younger physicians, thereby leading to increased number of reviews with high scores [8,11,23]. The associations among gender, total rating frequency, online presence, and rating score varied among studies [6,8,9,11,15,20,21,23-25].

Higher numbers of physician rating website profiles were seen for vascular surgeons with social media profiles, whether personal or departmental, but there was no statistical significance. Physicians with <14 or >40 years of experience were less likely to be rated. Small peaks were noted in the 20-24 years and 35-39 years of experience groups. This could be related to the similar trend seen in social media profiles. Gao et al also found that less experienced physicians had fewer ratings because they are still developing their practice and reputation [23]. Female vascular surgeons had fewer social media profiles and fewer physician rating website ratings compared to their male counterparts, which may be due to the younger age, and this finding has been reported in previous studies [11,20]. We found that physicians in practices with 10 or more surgeons had more ratings, but this finding has not been reported in previous studies. This could be related to large practices located in densely populated areas, which have higher number of ratings compared to the less densely populated regions reported in a recent study [16]. We did not examine metropolitan versus nonmetropolitan locations of practices ourselves because most of the academic affiliated practices were located in areas considered to be metropolitan.

The inconsistency in the physician ratings between physician rating websites and variable findings in factors predictive of better ratings is likely due to the low density of the ratings, leading to high susceptibility to outliers. Healthgrades, Vitals, WebMD, USNews&World Report, and Zocdoc were the only sites wherein the average number of ratings exceeded 5 for those rated. Vitals, WebMD, and Healthgrades were the only 3 websites, wherein over a quarter of the physicians were rated. These may be the better websites for vascular surgeons to focus on in a social media campaign. The large number of physician rating websites and directories—33 in 2010 and 28 in 2018—dilutes patient reviews [26,27]. Less than 5% of the physicians were rated on 56% (9/16) of the websites in our study. Less than 1% of the physicians were rated on 82% of the websites in the study of Lagu et al, and most of these sites were no longer accessible at the time of this study [26]. This rationalization and consolidation in the physician rating website marketplace will lead to an improved density of reviews.

Responding to a review is an important customer relationship exercise. No physician responded to any qualitative review in our study, including the 193 negative narrative reviews. In a study of the German physician rating website called “*Jameda,*” 1.58% of all the numeric ratings received responses while almost a third of the narrative reviews received responses from a physician [28]. Those physicians who responded to reviews had more “likes” and visits to the *Jameda* and had better ratings. Although these physicians were also more active on *Jameda,* improved rating and site travel may not be attributable to responding to patient reviews alone but they confirmed that active participation on physician rating websites has positive effects. Studies on response to patient reviews are limited in the medical field, but multiple studies in the hotel industry have found that responses to negative reviews can mitigate adverse effects [29-31].

Limited physician responses may be related to fear of HIPAA violation and offering an asynchronous medical opinion. Revealing patient information on social media without patient consent undermines patient trust and can lead to legal and civil actions [32]. However, most negative reviews are organizational issues outside the control of the providers, such as wait time, accessibility, and difficulty making appointment [10,26,33]. The best practices to actively respond to negative feedback is to respond offline or speak in the web-based platform in general terms, avoid confirming or denying the person as a patient, acknowledge the complaint issue, apologize, and provide an action plan [34,35]. Appropriate response to an active negative review can gain the trust of prospective patients auditing previous patient comments and reviews. In addition, the American Medical Association, along with others, recommend politely asking patients for reviews to dilute negative reviews since the majority will be positive [6,36].

Narrative reviews on physician rating websites have the potential of expanding the scope of quality measurement for providers. A recent study of Yelp reviews on hospitals has shown that patient reviews not only covered 7 out of the 12 categories included in the Hospital Consumer Assessment of Healthcare Providers and Systems but also consisted of additional 12 categories (Textbox 1) [37]. Studies have shown that physicians have used patient web-based reviews to improve patient care in areas of patient communication, scheduling process, and office workflow [38,39]. Narrative reviews on physician rating websites have the same potential of expanding the scope of quality measurements and aiding in quality improvement.

The limitations of physician rating websites include varied quality, limited accountability, and limited representativeness. In a global study on physician rating websites, the United States had a large number of physician rating websites but more considerable variation in the quality compared to European physician rating websites [40]. Anonymity, while beneficial for allowing uncensored speech, results in decreased accountability and false reviews such as negative reviews from competitors or self-written positive reviews [26]. While a third of patients provided their full name on Healthgrades, some appeared to be fake. This anonymity makes it difficult to validate their reviews or complaints on physician rating websites. The difficulty in validating and managing public responses is likely a reason that many hospitals do not engage in patient reviews. Furthermore, the web-based reviewer is not a random sample of the patient pool but rather an impressed or aggrieved party. There also exists demographic characteristics associated with physician-rating behavior and gender bias in perceptions of patient-physician interactions [25,41]. However, physician rating websites in the United States lack basic patient/reviewer demographics to allow further studies. Lastly, physician rating websites need to improve transparency by disclosing what enhancements are provided for sponsored profiles and clearly distinguish sponsored profiles not only during unspecific searches but also in specific searches and document it on the individual profiles.

Social media platforms have been used to advertise practice and attract referrals with relatively high returns on investment [42]. The physician rating website is an important part of these social media platforms. Third-party physician information websites, including the physician rating website, make up the majority of the top search results on Google [43]. Physician rating websites can attract patients by showing prior patient experiences and opinions, which can be exceedingly important in elective settings. Additionally, in concert with a well-designed social media strategy, physician rating websites can be a great platform to showcase a wide range of procedures that vascular surgeons perform alone or as assistance for other specialties. Given the findings in this study and a review of the current literature, we recommend the following physician rating website management strategies to improve value proposition: (1) focus on a limited number of physician rating websites (Vitals, Healthgrades, and WebMD), (2) assume the management profiles on physician rating websites to ensure the accuracy of information and allow physicians to receive instantaneous feedback, (3) invite patients to write reviews on these websites, and (4) develop a response strategy to reviews on the physician rating website chosen.

**Yelp domains that can supplement and inform traditional surveys of the patient experience of care.**

**Yelp domains**

- Cost of hospital visit
- Insurance billing
- Ancillary testing
- Facilities
- Amenities
- Scheduling
- Compassion of staff
- Family member care
- Quality of nursing
- Quality of staff
- Quality of technical aspects of care
- Specific type of medical care

### Conclusion

The representation of vascular surgeons on physician rating websites is varied, with the majority of the vascular surgeons represented only in half of the physician rating websites. The number of quantitative and qualitative reviews for vascular surgeons is low; therefore, no surgeon responded to any of the reviews. The activity of the vascular surgeons in this area of social media is low and reflects a small digital footprint that patients can reach and review. Healthgrades, Vitals, and WebMD are the most recommended physician rating platforms for vascular surgeons to focus on to promote and improve their practice.

## Appendix

Multimedia Appendix 1 Definitions of physician profiles.

Multimedia Appendix 2 List of physician rating websites included in our study.

## Abbreviations

HIPAA: Health Insurance Portability and Accountability Act
SAVS: Southern Association for Vascular Surgery
