# Supplementary material for: Digital Footprint of Academic Vascular Surgeons in the Southern United States on Physician Rating Websites: Cross-sectional Evaluation Study
Source: JMIR Cardio. 2021 Feb 24;5(1):e22975. doi: 10.2196/22975 (PMC8411431; doi:10.2196/22975)
Supplement: Multimedia Appendix 1 [file cardio_v5i1e22975_app1.docx]

Appendix 1. Definitions

Claimed profile: A profile is considered claimed if it is marked as “verified” or “claimed” or if it is not marked “unclaimed” when other profiles are.

Equivocal review: An “equivocal” review was defined as a review, where the entirety of the content did not contain any positive or negative phrase or if the review contains both positive and negative phrase.

General review sites: Refer to websites that included reviews on other sectors.

Healthcare review sites: Refers to websites only offering healthcare related information and healthcare related reviews.

Inaccurate profile: Profile with any inaccuracy in physician practice and demographic information.

Incomplete profile: Profile with less than 50% of required information (e.g. training, expertise) or lack any physician specific information besides practice location and office contact information.

Negative review: A “negative” review was defined by containing at least one or more negative comments without any positive comment.

Positive review: A “positive” review was defined as a review containing at least one or more positive comments without any negative comment about the provider or practice.

Southern Association for Vascular Surgery (SAVS):SAVS consists 13 southern states including: Alabama, Arkansas, Florida, Georgia, Kentucky, Louisiana, Maryland, Mississippi, North Carolina, South Carolina, Tennessee, Texas, Virginia, West Virginia, and Washington DC.

Sponsored profile: A profile is considered as sponsored if it is marked as “sponsored”. Profile with greater than 50% of required information provided were considered “complete”.
