# Supplementary material for: Digital Footprint of Academic Vascular Surgeons in the Southern United States on Physician Rating Websites: Cross-sectional Evaluation Study
Source: JMIR Cardio. 2021 Feb 24;5(1):e22975. doi: 10.2196/22975 (PMC8411431; doi:10.2196/22975)
Supplement: Multimedia Appendix 2 [file cardio_v5i1e22975_app2.docx]

Appendix 2. List of physician rating websites included in our study

| List of Physician Rating Website | |
| --- | --- |
|  | |
| Caredash | <https://www.caredash.com/> |
| DrScore | <http://www.drscore.com/index.cfm> |
| Healthcarereviews | <http://healthcarereviews.com/> |
| Healthgrades | <https://www.healthgrades.com/> |
| Insiderpages | <http://www.insiderpages.com/> |
| Local | <https://www.local.com/> |
| RateMDs | <https://www.ratemds.com/> |
| USNews&World Report | <https://health.usnews.com/doctors> |
| Vitals | <https://www.vitals.com/> |
| WebMD | <https://doctor.webmd.com/> |
| Wellness | <https://www.wellness.com/dir/search> |
| Yellowbook | [https://www.yellowbook.com/](https://www.yellowbook.com/us/miami-fl-33125-1610/) |
| Yellowbot | <https://www.yellowbot.com/> |
| Yelp | <https://www.yelp.com/> |
| YP – The Real Yellow Pages | <https://www.yellowpages.com/> |
| Zocdoc | <https://www.zocdoc.com/> |
